# Effect of Propolis on *Streptococcus mutans *Counts: An *in vivo *Study

**DOI:** 10.5005/jp-journals-10005-1180

**Published:** 2013-04-26

**Authors:** K Sundeep Hegde, Sham S Bhat, Ajay Rao, Shaniya Sain

**Affiliations:** Professor, Department of Pedodontics and Preventive Dentistry Yenepoya Dental College, Mangalore, Karnataka, India; Professor and Head, Department of Pedodontics and Preventive Dentistry, Yenepoya Dental College, Mangalore, Karnataka, India; Reader, Department of Pedodontics and Preventive Dentistry, Yenepoya Dental College, Mangalore, Karnataka, India; Postgraduate Student, Department of Pedodontics and Preventive Dentistry, Yenepoya Dental College, Mangalore, Karnataka, India

**Keywords:** Propolis, *Streptococcus mutans*count, Saliva

## Abstract

Propolis, a natural antibiotic, is a resinous substance that honey bees (*Apis mellifera*) produce. The main chemical classes present in propolis are flavonoids, phenolics and other various aromatic compounds.

**Aim:** To evaluate the antibacterial action of propolis on the concentration of *Streptococcus mutans *colonizing the oral cavity of children.

**Materials and methods:** Thirty children performed the rinses, with no other changes in their oral hygiene and dietary habits. Saliva was collected at two time points: Before using the product, 1 hour after the rinse.

**Results:** Paired t-test was used for analysis of the results. A reduction in the concentration of *Streptococcus mutans *was observed in samples collected after use of the extract. There was a reduction in *Streptococcus mutans *count when compared to samples obtained in baseline. Significant reductions were seen at the end of 1 hour. The result was statistically significant. There were no side effects in soft and hard tissues of mouth.

**Conclusion and clinical implication:** The propolis possesses *in vivo *antimicrobial activity against *Streptococcus mutans *present in the oral cavity and might be used as a measure to prevent dental caries.

**How to cite this article:** Hegde KS, Bhat SS, Rao A, Sain S. Effect of Propolis on *Streptococcus mutans* Counts: An *in vivo* Study. Int J Clin Pediatr Dent 2013;6(1):22-25.

## INTRODUCTION

In the new era of globalization there is an evident paradigm shift in health care approaches. The critical aspect of focus and research is toward evolving innovative green perceptions in health care research and practices. The basis of these movements is anchored towards producing tangible process linked products that could be applicable on a global scale and carries with itself a label–Eco friendly. This line of thought has lead therapeutic research to experiment with medicinal properties of various plants and to evolve pharmaceutical products.

In similar context, propolis is a resinous substance. The word propolis (Russian Penicillin) is derived from the Greek word ‘pro’ before, polis ‘city’ or defender of the city. Honey bees (*Apis mellifera*) collect the resin from cracks in he bark of trees and leaf buds. It is masticated, salivary nzymes are added and the partially digested material is mixed with bee wax and used by bees to seal holes in their honeycombs, smooth out the internal walls and protect the entrance against external agents and contaminants.^1^ Propolis is composed of 50% resin and vegetable balsam, 30% wax, 10% essential and aromatic oils, 5% pollen and 5% various other substances, including organic debris depending on the place and time of collection.^2,3^ It is a natural antibiotic. The medicinal properties are due to the flavonoids, phenolics and various aromatic compounds. Flavonoids have antibacterial, antifungal, antiviral, antioxidant and anti-inflammatory proprieties. Galangin, pinocembrin and pinostrobin are known as the most effective flavonoids agents against bacteria. Ferulic acid and caffeic acid also contribute to the bactericidal action of propolis.^4^

Propolis has been widely used for clinical trials in dentistry for various purposes and seems to be promising. As an anti-inflammatory agent, propolis is shown to inhibit synthesis of prostaglandins, activate the thymus gland, aid the immune system by promoting phagocytic activity, stimulate cellular immunity and augment healing effects on epithelial tissues.^5-7^ Propolis also contains iron and zinc that are important for the synthesis of collagen.

Potential uses in dentistry are wound healing, storage media following avulsion,^8^ a pulp capping agent, intracanal rrigant, intracanal medicament, mouth rinse, cariostatic agent, n dentinal hypersensitivity, in treatment of periodontitis, has effect on *Candida albicans*, in treatment of denture stomatitis and has effect on recurrent aphthous stomatitis.^9^

Dental caries is the most prevalent disease affecting humans, and its susceptibility is much higher in childhood. During the initial phase of caries, *Streptococcus mutans* is the most frequently associated microorganism. In addition to its ability to adhere to teeth and survive in acid environment, *Streptococcus mutans* is transmissible, as first demonstrated by Keyes in his trials.^10^

## AIM

To evaluate the antibacterial action of propolis on *Streptococcus mutans* colonizing the oral cavity of children.

## OBJECTIVE

The objective of the study was to evaluate the *in vivo* antimicrobial activity of an extract prepared with propolis when used as mouth rinse on the colony-forming units of *Streptococcus mutans* present in the oral cavity of children.

## MATERIAL AND METHOD

### Determination of Antibacterial Efficacy

**Fig. 1:**
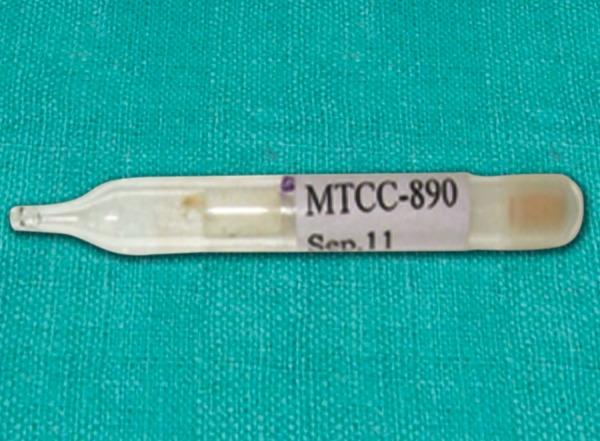
MTCC-890 strain

**Fig. 2:**
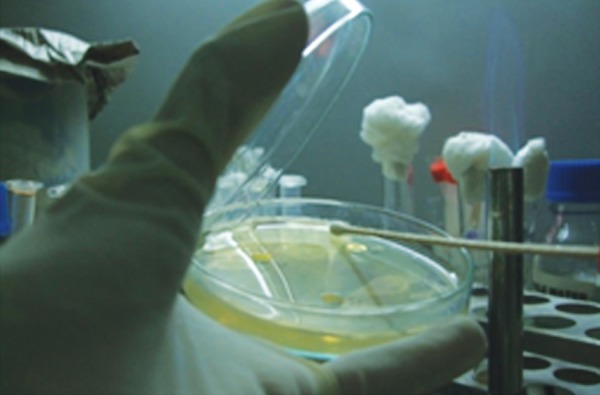
Determination of antibacterial efficacy

*Streptococcus mutans* MTCC 890 strains (Fig. 1) were used for the study. Serial dilutions of propolis (20, 10, 5, 3 and 2.5%) were used. *Streptococcus mutans* culture was swabbed evenly onto Trypticase soy agar (TSA) media (Fig. 2) and wells

### Preparation of Propolis

**Fig. 3:**
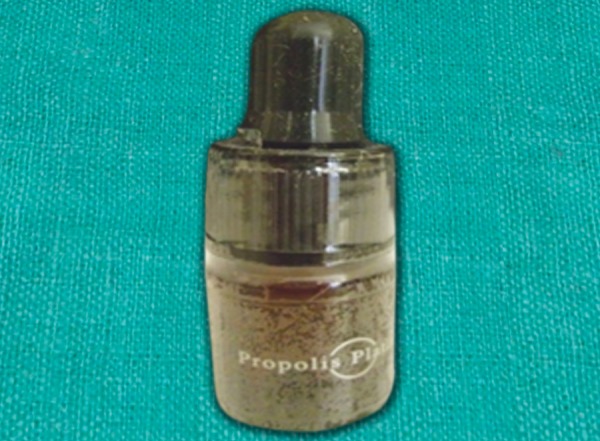
Commercially available propolis extract

**Fig. 4:**
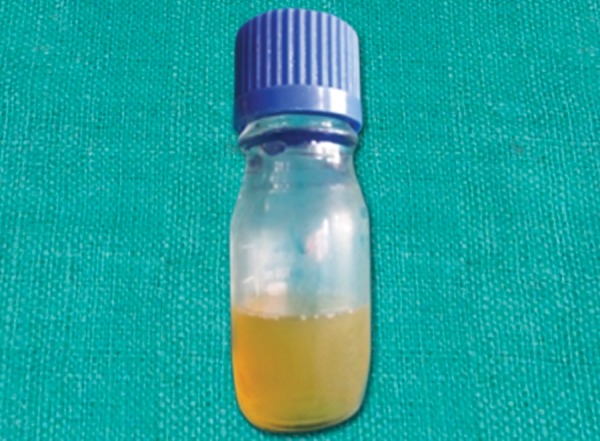
Diluted propolis extract

Five percent propolis, commercially available as propolis platinum [K-Link Healthcare (India) Pvt Ltd Chennai] (Fig. 3) was diluted in sterile water (5 ml diluted in 90 ml of sterile water) and was used for the study (Fig. 4).

**Fig. 5:**
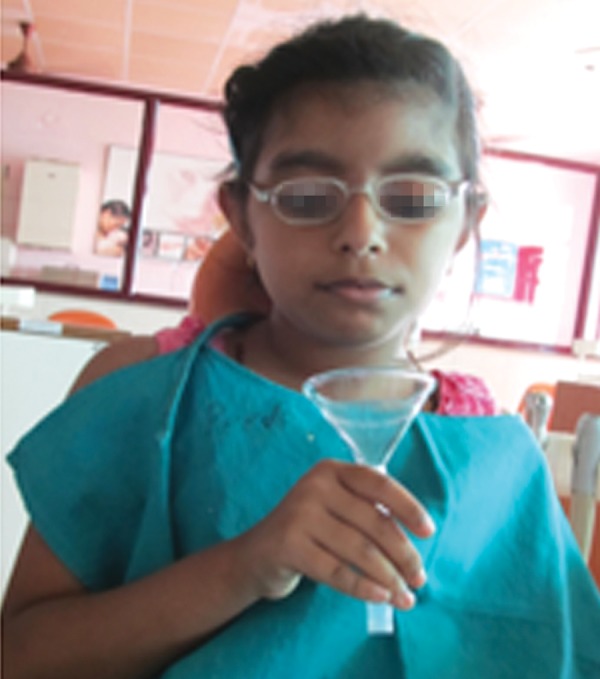
Collection of saliva

Thirty children of both sexes, ranging in age from 5 to 10 years were enrolled for the study with the consent of parents. First samples of whole saliva were collected into sterile collection vials (3 ml on the average) and it was seeded in the laboratory (Fig. 5).

**Fig. 6:**
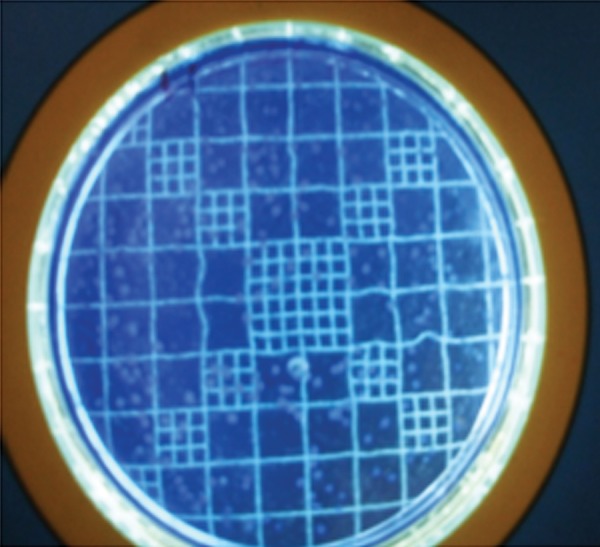
Colony counting grid

**Fig. 7:**
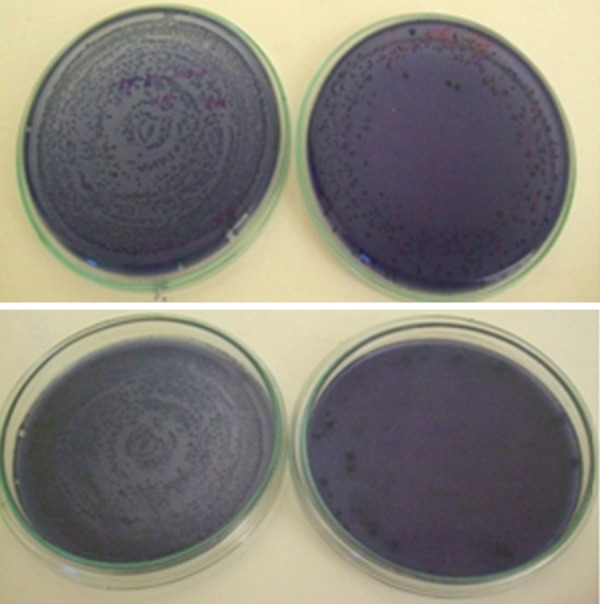
Pre and post samples with *Streptococcus mutans*

The children were then asked to rinse their mouth with 3 ml of the diluted propolis extract solution for 1 minute and a second saliva samples was collected 1 hour later in the same way as described for the first sample. The volunteers performed the mouth rinses with no other changes in their oral hygiene, dietary habits and day-to-day practices. The microbiological analysis was carried out using the selective *Streptococcus mutans* media. It permits semiquantitative analysis of this microorganism in salivary samples. Finally colony-forming units were counted (Figs 6 and Figs 7).

## RESULT

Minimum inhibition concentration was seen to be 5%. Of the 30 saliva samples collected from the 30 volunteers, prebacterial counts ranged from 800 to 91,00,000 CFU/ml saliva (Table 1).

**Graph 1:**
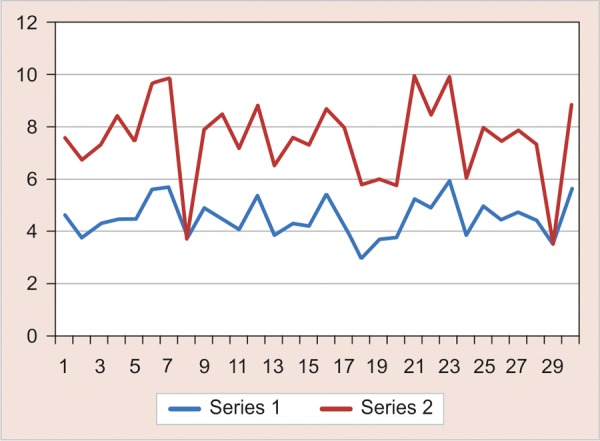
Red line indicates pre and blue lines indicates post results

Statistical analysis was done by the paired t-test. The results showed a significant difference in the number of *S. mutans* between collections 1 and 2 (mean ± SD: 1.1597 ± 0.8560; t = 2.045 and p < 0.05) i.e. an effect of propolis on bacterial growth both after the beginning (collection 1) and at the end of treatment (collection 2). These results indicate a reduction in the number of *S. mutans *(Graph 1). Analysis of variance to determine the relationship between the number of mouth rinses and bacterial counts indicated a significant difference.

**Table 1:** **Table 1:** Pre and post counts of *Streptococcus mutans*

*No.*	*Pre*	*Post (1 hour)*	*Log 1*	*Log 2*	*Log 1-Log 2*
1.	40000	2600	4.602	3.414	1.188
2.	6000	4000	3.778	3.602	0.1761
3.	20000	1500	4.301	3.176	1.125
4.	28000	14000	4.447	4.146	0.301
5.	30000	1000	4.477	3	1.447
6.	400000	30000	5.602	4.477	1.125
7.	500000	20000	5.698	4.301	1.397
8.	5000	0	3.698	0	3.698
9.	80000	4000	4.903	3.602	1.301
10.	32000	2800	4.505	4.447	0.103
11.	11000	5000	4.041	3.698	0.343
12.	270000	10000	5.431	4	1.431
13.	7000	2500	3.845	3.397	0.448
14.	20000	1300	4.301	3.113	1.188
15.	16000	7200	4.204	3.857	0.347
16.	300000	23000	5.477	4.361	1.116
17.	19000	8000	4.278	3.903	0.375
18.	800	800	2.903	2.903	0.000
19.	5000	400	3.698	2.602	1.096
20.	6000	200	3.778	2.301	1.477
21.	180000	90000	5.255	4.954	0.301
22.	86000	10000	4.934	4	0.934
23.	910000	43000	5.959	4.633	1.326
24.	6200	530	3.792	2.724	1.068
25.	98000	3000	4.991	3.477	1.514
26.	29000	1300	4.462	3.113	1.349
27.	60000	2300	4.778	3.361	1.417
28.	30000	1000	4.477	3	1.477
29.	3000	0	3.477	0	3.477
30.	400000	2200	5.602	3.342	2.260

### DISCUSSION

Majority of the samples showed a decrease in the colonies. A total of 90% showed reduction in bacterial load. In 6.6% there were no colony-forming units after mouth rinse. A total of 3.4% showed no reduction after rinsing with mouthwash. A significant reduction in the number of colonies in the samples is the result of the effect of the propolis extract on bacterial growth. Samples that showed no change in the number of bacteria between collections might have been influenced by overlapping factors, such as a delayed peak formation of colonies, i.e. more than 2 hours after the meal, together with the short period for an effective action and the transmissible nature of *S. mutans*.^11,12^ There were no side effects in soft and hard tissues of mouth.

*Streptococcus mutans* is not the only one organism which causes the disease. The etiology of dental caries clearly points out that dental caries is multifactorial. Many other microbial agents along with time, dietary factors and host factors results in caries.^13^

Studies had demonstrated that host binding characteristics are as important as the characteristics of bacterial adhesion in the process of colonization. It was suggested that salivary amylase may show the best binding to *S. mutans*. It was also seen that bacterial interactions have a key role in colonization.^14^

Propolis has activity against Gram-positive, Gram-negative organisms and even against *Candida*. Certain chemical components of propolis act on the cell wall of microorganisms causing functional and structural damages. It has mucoprotective effect so can be used efficiently in the oral cavity.^15^

Within the philosophy of health promotion, the extract of propolis may represent a new option showing long-term beneficial effects. Further clinical studies with large samples, long-term follow-up and comparison with conventional mouthwash is required.

## CONCLUSION

The propolis possesses *in vivo* antimicrobial activity against *S. mutans* present in the oral cavity and might be used as an alternative measure to prevent dental caries.
